# Impact of polypharmacy and comorbidity on survival and systemic parenteral treatment administration in a cohort of hospitalized lung-cancer patients

**DOI:** 10.1186/s12885-023-10939-7

**Published:** 2023-06-23

**Authors:** Hélène Pluchart, Sébastien Bailly, Sébastien Chanoine, Denis Moro-Sibilot, Pierrick Bedouch, Anne-Claire Toffart

**Affiliations:** 1 Pôle pharmacie, CHU Grenoble Alpes, Grenoble, France; 2 Université Grenoble Alpes, Grenoble, France; 3 Univ. Grenoble Alpes, CNRS, Grenoble INP, CHU Grenoble Alpes, TIMC, Grenoble, 38000 France; 4 Univ. Grenoble Alpes, Inserm, CHU Grenoble Alpes, HP2, Grenoble, 38000 France; 5 Institute for Advanced Biosciences, UGA/INSERM U1209/CNRS 5309, Université Grenoble Alpes, Grenoble, France; 6 Clinique de Pneumologie, Unité d’Oncologie Thoracique, Pôle Thorax et Vaisseaux, CHU Grenoble Alpes, Grenoble, France

**Keywords:** Lung cancer, Comorbidity, Polypharmacy, Survival

## Abstract

**Background:**

Although polypharmacy has been described among cancer patients, very few studies have focused on those with lung cancer. We aimed to assess whether polypharmacy and comorbidity have an impact on systemic parenteral treatment administration and survival among lung-cancer patients.

**Methods:**

In this retrospective monocenter cohort study, we included patients hospitalized in thoracic oncology for the first time between 2011 and 2015. The Elixhauser score was used to assess comorbidity and polypharmacy was estimated with a threshold of at least five prescribed medications. The Fine and Gray competitive risk model was used to estimate the impact of polypharmacy and comorbidity on systemic parenteral treatment administration within the first two months of hospitalization. The effect of comorbidity and polypharmacy on overall survival was evaluated by Cox proportional hazards analysis.

**Results:**

In total, 633 patients were included (71% men), with a median age of 66 years. The median Elixhauser score was 6 and median overall survival was four months. Among the patients, 24.3% were considered to be receiving polypharmacy, with a median number of medications of 3, and 49.9% received systemic parenteral treatment within two months after hospitalization. Severe comorbidity (Elixhauser score > 11), but not polypharmacy, was independently associated with a lower rate of systemic parenteral treatment prescription (SdHR = 0.4 [0.3;0.6], p < 0.01) and polypharmacy, but not a high comorbidity score, was independently associated with poorer four-month survival (HR = 1.4 [1.1;1.9], p < 0.01)

**Conclusions:**

This first study to evaluate the consequences of comorbidity and polypharmacy on the care of lung-cancer patients shows that a high comorbidity burden can delay systemic parenteral treatment administration, whereas polypharmacy has a negative impact on four-month survival.

## Background

With a median age at diagnosis of 70 years [1], lung cancer mainly concerns the elderly. In addition to their being diagnosed at advanced stages [2, 3], lung-cancer patients are mostly diagnosed with prior comorbidities due to smoking [4, 5]. Comorbidities negatively influence lung cancer survival and have consequences on treatment strategies [4, 6, 7].

In parallel, comorbidities are generally associated with polypharmacy because they require the prescription of medications. Moreover, anticancer treatment can lead to side effects and symptomatic treatment prescriptions, with frequent polypharmacy [8]. The resulting consequences can be important. For example, polypharmacy for chronic comorbid conditions can induce drug-drug interactions with anticancer treatment [9] or lead to a higher risk of side effects [10].

Polypharmacy has been reported to be a potential risk factor for death [11]. We aimed to determine whether comorbidity and polypharmacy have an impact on systemic parenteral treatment administration and survival, specifically for lung cancer patients.

## Methods

### Population

Patients hospitalized in the Thoracic Oncology Unit of Grenoble University Hospital from 2011 to 2015 were included. The design of this study has been described in an earlier publication [12]. Lung-cancer patients were included at their first hospitalization during the study period. Ethics committee approval for the study was obtained on September 1, 2021 (Comité d’Ethique des Centres d’Investigation Clinique Rhône-Alpes-Auvergne, Clermont-Ferrand, IRB 5891).

### Objectives

The primary objective was to assess the impact of polypharmacy and comorbidities on systemic parenteral treatment administration after the first hospitalization in thoracic oncology. The primary outcome was the time to systemic parenteral treatment administration after the date of hospitalization (within 2 months). Two months was chosen as the cut-off because it corresponds to the postponement of systemic parenteral treatment administration due to toxicity and delays beyond this timepoint are considered to be due to reasons other than systemic parenteral treatment -related toxicity.

As a secondary objective, we assessed the impact of polypharmacy and comorbidity on overall survival, estimated as the time between the day of hospitalization and the date of last follow-up (cut off at median overall survival).

### Data collection

The database contains information on individuals including age, gender, lung cancer tumor (T), node (N), and metastasis (M) (TNM) staging, histological type, performance status at the first case presentation in multidisciplinary concertation meetings, the Elixhauser score for comorbidities [13], and every medication administered throughout the hospitalization. Elixhauser score is a comorbidity score that weighs comorbidity burden. It takes into account 21 conditions. The score range is from − 19 to 89 (high score means high comorbidity burden).

Polypharmacy was defined as > 5 administered medications. A medication was considered if the patient had at least one dose. Each medication was translated using its fourth, third, and second level in the Anatomical Therapeutic Chemical (ATC) Classification System. ATC Classification System is an international tool to classify drugs and can be used in research. It is controlled by the World Health Organization Collaborating Centre for Drug Statistics Methodology. Anticancer treatment, anti-emetic drugs, blood substitutes, and perfusion solutions were not studied because they were not prescribed for chronic comorbidities.

Survival data were obtained from our district cancer registry, including the date of the last follow-up and vital status at the last follow-up.

### Statistical analyses

For descriptive analyses, quantitative variables are expressed as medians [Interquartile range] and qualitative variables as n (%).

Median overall survival depending on comorbidity and polypharmacy was assessed by estimating the probability of survival using the Kaplan Meier estimator. Survival curves were compared using Log-rank tests. A Cox proportional hazards regression model was used to identify factors associated with survival. Univariable and multivariable analyses were performed. Proportional hazards assumptions were verified using the Martingale method. Only covariables with p < 0.2 in univariable analysis were retained for multivariable analysis.

The impact of polypharmacy and comorbidities on systemic parenteral treatment administration was determined using the Fine and Gray competing risk regression model to account for the competitive risk of death [19]. Sub-distributed hazard ratios (SdHRs) and their confidence intervals [CI95] were calculated.

Gender, metastasis, histological type, polypharmacy, the Elixhauser score, age at hospitalization, and age at diagnosis were included as covariates. A severe Elixhauser threshold score was defined as the third interquartile range of the median score. Performance status was not included because of missing data. For the secondary objective, TNM stage, time of diagnosis (after of before hospitalization), and time to systemic parenteral treatment (used as a time-dependent variable) were also included.

All statistical analyses were performed using SAS 9.4 for Windows (SAS Institute, Inc., Cary, NC, USA). A P-value < 0.05 was considered significant.

## Results

### Patient characteristics

In total, 633 patients were included, with a median age of 66 years (Table 1). They were mostly men (71%) and most patients had metastatic lung cancer (428 [74.2%)]). According to the Elixhauser score, weight loss and chronic pulmonary disease were the two most common comorbidities in the population (345 [54.5%] and 91 [14.4%] patients, respectively).

**Table 1:** Description of the population

	Population (n = 633)
Patients’ characteristics and comorbidities	
Age (years)	66 [58; 73]
Gender = Men	540 (71)
Elixhauser Score	6.0 [2.0; 11.0]
Two most common comorbidities according to the Elixhauser score	
Weight loss	345 (54.5)
Chronic pulmonary disease	91 (14.4)
Performance status at the first presentation in multidisciplinary concertation meetings*	
• PS 0–1	212 (53.1)
• PS 2	126 (31.6)
• PS 3–4	61 (15.3)
Metastasis	428 (74.2)
Histological type	
• Adenocarcinoma	295 (46.6)
• Squamous-cell carcinoma	103 (16.3)
• Undifferentiated	67 (10.6)
• SCLC	132 (20.9)
• Other	36 (5.7)
**Medication**	
Number of medications	3 [2–4]
Polypharmacy (excluding anticancer treatment, anti-emetic drugs, blood substitutes, and perfusion solutions)	154 (24.3)
**Outcomes/survival**	
Overall survival (months)	4 [1; 11]
Patients having systemic parenteral treatment within two months	316 (49.9)
Death within two months without receiving systemic parenteral treatment	175 (27.6)
Alive two months without systemic parenteral treatment	142 (22.4)
Time to systemic parenteral treatment administration (days)	13 [5–29]
Time to systemic parenteral treatment administration for patients receiving treatment within two months (n = 316) (days)	11 [4–22]

Quantitative variables are expressed as medians [Interquartile range], qualitative variables are expressed as n (%)

*Missing data: n = 234

The median number of medications was 3 [IQ25% 2- IQ75% 4] and the number of patients with polypharmacy was 154 (24.3%).

### Medication description

Among the medications, the ten most prescribed ATC codes, according to the second level of the ATC classification system, are presented in Table 2. Approximately 4 of 10 patients received analgesics and corticosteroids for systemic use. A quarter of patients also received mineral supplements, whereas drugs for constipation and antibacterial and antithrombotic agents were prescribed for 2 of 10 patients.

**Table 2:** The ten most prescribed medications according to the second level of the ATC Classification System

ATC Code (according to second level of ATC Classification System)*	Population (n = 633)
N02 (Analgesics)	265 (41.9)
H02 (Corticosteroids for systemic use)	258 (40.8)
A12 (Mineral supplements)	162 (25.6)
A06 (Drugs for constipation)	129 (20.4)
J01 (Antibacterials for systemic use)	118 (18.6)
B01 (Antithrombotic agents)	112 (17.7)
V03 (All other therapeutic products)	74 (11.7)
A10 (Drugs used in diabetes)	57 (9.0)
R03 (Drugs for obstructive airway diseases)	36 (5.7)
R06 (Antihistamines for systemic use)	40 (6.3)

Qualitative variables are expressed as n (%)

*Excluding anticancer treatment, antiemetics, blood substitutes and perfusion solutions

### Factors associated with systemic parenteral treatment administration and survival

Median overall survival was four months and the median Elixhauser score was 6.0 [IQ25% 2.0; IQ75%11.0] (Table 1). By two months within hospitalization, 316 (49.9%) patients received systemic parenteral treatment, 175 (27.6%) died without receiving systemic parenteral treatment, and 142 (22.4%) were alive without receiving systemic parenteral treatment. Systemic parenteral treatment was prescribed and administered after a median of 11 [IQ25% 4 ; IQ75% 22] days for patients receiving systemic parenteral treatment within two months.

Consideration of the SdHR with the competing event (death) for the outcome of systemic parenteral treatment administration showed that only severe comorbidity (Elixhauser score > 11) led to a lower rate of systemic parenteral treatment prescription (SdHR = 0.4 [0.3;0.6], p < 0.01)) (Table 3).

**Table 3:** SdHR for receiving systemic parenteral treatment considering death as a competing event among the population (n = 633)

	Univariable analysis						Multivariable analysis*		
	**sdHR**		**CI95**			**P-value**	**sdHR**	**CI95**	**P-value**
**Elixhauser**									
< 0 < x ≤ 5	1						1		
6 ≤ x ≤ 11	0.8		0.6-1.0			0.08	0.8	0.7–1.1	0.19
> 11	0.4		0.3–0.6			< 0.01	0.4	0.3–0.6	< 0.01
**Polypharmacy**	1.3		1.0-1.6			0.06	1.2	1.0-1.6	0.10

Quantitative variables are expressed as medians [Interquartile range]

*Adjusted for the presence of metastasis, gender, and histological type

Overall survival according to the Elixhauser score was 11.4 [IQ25% 2.0; IQ75% 17.0] months for an Elixhauser score between 0 and 5, 6.8 [1.0; 8.0] months for an Elixhauser score between 6 and 11, and 6.7 [IQ25% 1.0 ; IQ75% 8.0] months for an Elixhauser score > 11. Only polypharmacy (threshold of 5 medications) was a negative prognostic factor of survival in multivariate analysis (HR = 1.4 [1.1–1.9], p < 0.01) (Table 4).

**Table 4:** Adjusted hazard ratio of four-month survival among the population (n = 633)

		Univariable analysis			Multivariable analysis*		
	**Overall survival**	**HR**	**CI95**	**P-value**	**HR**	**CI95**	**P-value**
**Elixhauser**							
< 0 < x ≤ 5 (n = 234)	11.4 [2.0;17.0]	1			1		
6 ≤ x ≤ 11 (n = 245)	6.8 [1.0;8.0]	1.5	1.1–1.9	< 0.01	1.2	0.9–1.6	0.14
> 11 (n = 154)	6.7 [1.0;8.0]	1.8	1.3–2.4	< 0.01	1.1	0.8–1.5	0.64
**Polypharmacy**		1.2	1.0-1.6	0.09	1.4	1.1–1.9	< 0.01

*Adjusted for the presence of metastasis, TNM stage, gender, histological type, time of diagnosis, and time to systemic parenteral treatment as a time-dependent variable

## Discussion

This study shows that severe comorbidity (assessed by the Elixhauser score) is associated with a lower rate of systemic parenteral treatment prescription within two months of hospitalization, whereas polypharmacy is an independent prognostic factor of death within four months of first hospitalization.

We found polypharmacy to be present for 24.3% of patients, which is lower than that reported in previous studies concerning various cancer types. Previous studies found polypharmacy to range from 33 to 59.9% among lung-cancer patients [14, 15]. In these studies, the estimated number of drugs was based on the number of unique prescriptions in the month prior to the diagnosis or as any therapeutic drug used to manage a comorbid condition outside of lung cancer between the last visit and day 1 of the oncology treatment. However, these studies did not indicate whether they counted, for example, anti-emetic drugs. In another recent study, the median number of prescribed medications during the six-month period before chemotherapy administration for lung cancer patients was 11 [16]. Among elderly patients with any type of cancer, Nightingale et al. reported a mean number of medications of nine associated with a prevalence of polypharmacy of 41% [17]. In this study, patient medications were assessed during a pharmacist-patient session to which they had to bring all medications (prescription, nonprescription, herbals, and supplements) they had at home. We did not have access to data on self-medications or herbal medications in our study. The lower rate of polypharmacy found in our cohort could be explained by the fact that complementary and alternative medicines are widely used by lung-cancer patients (reported to be from 30 to 50% of patients [18, 19]). In addition, only prescribed medications were considered in our study, excluding anticancer treatment, anti-emetics, blood substitutes, and perfusion solutions. Within two months after hospitalization, 175 (27.6) patients died without receiving systemic parenteral treatment. Given the poor prognosis, certain medications were probably stopped in light of the benefit-risk balance for the patient.

Polypharmacy has been shown to be possibly related to greater chemotherapy toxicity [10]. Here, we did not have information on the potential side effects after systemic parenteral treatment. Sud et al. reported the impact of polypharmacy on increased hospitalization rates, with toxicity rather than polypharmacy leading to the discontinuation of anticancer therapy among those aged 80 and over [20], whereas Lu-Yao et al. showed polypharmacy to be a predictive factor of inpatient hospitalization after chemotherapy administration [16]. Another explanation may be due to the study design. We estimated polypharmacy by the number of medications received. Some may have been prescribed as symptomatic treatment due to an adverse event, increasing the number of medications received. It is possible that the burden of comorbidity itself influences the physician’s decision whether or not to prescribe systemic parenteral treatment rather than the number of medications.

Patients with severe comorbidity, defined by an Elixhauser score > 11, were significantly less likely to receive systemic parenteral treatment within two months after their first hospitalization, with no impact of polypharmacy. One explanation may be that there is probably no treatment impact on systemic parenteral treatment prescription for comorbidities that are well managed by treatment. The impact of comorbidities on the prescription of treatment was expected, as concomitant comorbid conditions potentially lead to increased toxicity of systemic parenteral treatment [21]. The 10 most prescribed drugs that we found, according to their second level ATC code, are consistent with those reported in the literature [4, 5]. The comorbidity burden has been reported to decrease the prescription of systemic parenteral treatment in colorectal, breast, and bladder cancer [22–24]. Few studies in lung cancer are available but they also reported an association between a high comorbidity burden and systemic parenteral treatment administration [25–27].

In terms of the impact of polypharmacy and comorbidity on survival, our findings are consistent with those of Hakozaki et al., who reported an association between polypharmacy and lower overall survival but not progression-free survival for lung cancer patients receiving immune oncology treatments [15]. The impact of comorbidity on survival has also been reported to be less important for patients with a poor prognosis. Indeed, Piccirillo et al. showed that comorbidity was prognostically of less importance for diseases with a short-term prognosis, such as lung cancer [28].

A strength of our data is the large sample size, associated with a robust statistical analysis, provided by the use of several datasets with a deterministic linkage. Our study, however, also had several limitations. Because of the study design, we did not consider drug-drug interactions. In addition, no data on side effects after systemic parenteral treatment were available. Finally, we could not consider self-medication or complementary or alternative medicines due to the retrospective monocenter design of our study.

## Conclusion

In this study, severe comorbidity had an impact on systemic parenteral treatment prescription, whereas polypharmacy was associated with poorer survival. Access to the National Health Insurance Cross-Scheme Information System could be useful to better describe the link between polypharmacy, comorbidity, and their consequences on anticancer treatment and survival, and prospective studies should be performed.

## Data Availability

The datasets generated and/or analysed during the current study are not publicly available due to privacy restrictions but are available from the corresponding author on reasonable request. The description of their content and how it has been obtained is described in the protocol cited above (12).

## Abbreviations

ATC: Anatomical Therapeutic Chemical
SdHRs: Sub-distributed hazard ratios
TNM: Tumor, Nodes and Metastasis

## References

[1] Noone AM, Howlader N, Krapcho M, Miller D, Brest A, Yu M et al. SEER Cancer Statistics Review, 1975–2015, National Cancer Institute [Internet]. [cited 2018 Jul 30]. Available from: https://seer.cancer.gov/csr/1975_2015/sections.html.

[2] Jemal A, Siegel R, Xu J, Ward E. Cancer statistics, 2010. CA Cancer J Clin. 2010 Oct;60(5):277–300. 10.3322/caac.20073 20610543

[3] Howlader N, Noone AM, Krapcho M, Garshell J, Miller D, Altekruse SF et al. SEER Cancer Statistics Review, 1975–2011, National Cancer Institute [Internet]. [cited 2018 Jul 10]. Available from: https://seer.cancer.gov/archive/csr/1975_2011/.

[4] Islam KMM, Jiang X, Anggondowati T, Lin G, Ganti AK. Comorbidity and Survival in Lung Cancer Patients. Cancer Epidemiol Biomarkers Prev. 2015 Jul;24(7):1079–85. 10.1158/1055-9965.EPI-15-0036 26065838

[5] Edwards BK, Noone A-M, Mariotto AB, Simard EP, Boscoe FP, Henley SJ et al. Annual Report to the Nation on the status of cancer, 1975–2010, featuring prevalence of comorbidity and impact on survival among persons with lung, colorectal, breast, or prostate cancer. Cancer. 2014 May 1;120(9):1290–314. 10.1002/cncr.28509 PMC3999205 24343171

[6] Leduc C, Antoni D, Charloux A, Falcoz P-E, Quoix E. Comorbidities in the management of patients with lung cancer. Eur Respir J. 2017;49(3). 10.1183/13993003.01721-2016 28356370

[7] Morishima T, Matsumoto Y, Koeda N, Shimada H, Maruhama T, Matsuki D, et al. Impact of comorbidities on survival in gastric, colorectal, and Lung Cancer Patients. J Epidemiol. 2019 Mar;5(3):110–5. 10.2188/jea.JE20170241 PMC6375811 30012908

[8] Nightingale G, Skonecki E, Boparai MK. The impact of polypharmacy on patient outcomes in older adults with Cancer. Cancer J. 2017 Aug;23(4):211–8. 10.1097/PPO.0000000000000277 28731943

[9] Alkan A, Yaşar A, Karcı E, Köksoy EB, Ürün M, Şenler F et al. Severe drug interactions and potentially inappropriate medication usage in elderly cancer patients. Supportive Care in Cancer 2016 Sep 12;25:229–36. 10.1007/s00520-016-3409-6 27619388

[10] Mohamed MR, Ramsdale E, Loh KP, Arastu A, Xu H, Obrecht S, et al. Associations of Polypharmacy and Inappropriate Medications with adverse outcomes in older adults with Cancer: a systematic review and Meta-analysis. Oncologist. 2020 Jan;25(1):e94–108. 10.1634/theoncologist.2019-0406 PMC6964156 31570516

[11] Leelakanok N, Holcombe AL, Lund BC, Gu X, Schweizer ML. Association between polypharmacy and death: a systematic review and meta-analysis. J Am Pharmacists Association. 2017 Nov;57(6):729–738e10. 10.1016/j.japh.2017.06.002 28784299

[12] Pluchart H, Bailly S, Fauconnier J, Delafosse P, Chanoine S, Dumas I et al. Study protocol to assess polypharmacy and comorbidities in lung cancer. Respiratory Med Res. 2021 Sep;100861. 10.1016/j.resmer.2021.100861 34662777

[13] van Walraven C, Austin PC, Jennings A, Quan H, Forster AJ. A modification of the Elixhauser comorbidity measures into a point system for hospital death using administrative data. Med Care. 2009 Jun;47(6):626–33. 10.1097/MLR.0b013e31819432e5 19433995

[14] Lund JL, Sanoff HK, Peacock Hinton S, Muss HB, Pate V, Stürmer T. Potential medication-related problems in older breast, Colon, and Lung Cancer Patients in the United States. Cancer Epidemiol Biomarkers Prev. 2018 Jan;27(1):41–9. 10.1158/1055-9965.EPI-17-0523 PMC5760326 28978563

[15] Hakozaki T, Hosomi Y, Shimizu A, Kitadai R, Mirokuji K, Okuma Y. Polypharmacy as a prognostic factor in older patients with advanced non-small-cell lung cancer treated with anti-PD-1/PD-L1 antibody-based immunotherapy. J Cancer Res Clin Oncol. 2020 Oct;146(10):2659–68. 10.1007/s00432-020-03252-4 PMC11804739 32462298

[16] Lu-Yao G, Nightingale G, Nikita N, Keith S, Gandhi K, Swartz K, et al. Relationship between polypharmacy and inpatient hospitalization among older adults with cancer treated with intravenous chemotherapy. J Geriatr Oncol. 2020 May;11(4):579–85. 10.1016/j.jgo.2020.03.001 PMC8261864 32199776

[17] Nightingale G, Hajjar E, Swartz K, Andrel-Sendecki J, Chapman A. Evaluation of a pharmacist-led medication assessment used to identify prevalence of and associations with polypharmacy and potentially inappropriate medication use among ambulatory senior adults with cancer. J Clin Oncol. 2015 May 1;33(13):1453–9. 10.1200/JCO.2014.58.7550 25800766

[18] Micke O, Buntzel J, Kisters K, Schafer U, Micke P, Mucke R (2010). Complementary and alternative medicine in lung cancer patients: a neglected phenomenon?. Front Radiat Ther Oncol.

[19] Wyatt GK, Friedman LL, Given CW, Given BA, Beckrow KC. Complementary therapy use among older cancer patients. Cancer Pract. 1999 Jun;7(3):136–44. 10.1046/j.1523-5394.1999.07305.x 10352076

[20] Sud S, Lai P, Zhang T, Clemons M, Wheatley-Price P. Chemotherapy in the oldest old: the feasibility of delivering cytotoxic therapy to patients 80years old and older. J Geriatric Oncol. 2015 Sep;6(5):395–400. 10.1016/j.jgo.2015.07.002 26278886

[21] Sarfati D, Koczwara B, Jackson C. The impact of comorbidity on cancer and its treatment: Cancer and Comorbidity. CA: a Cancer Journal for Clinicians. 2016 Jul;66(4):337–50. 10.3322/caac.21342 26891458

[22] Lee L, Cheung WY, Atkinson E, Krzyzanowska MK. Impact of Comorbidity on Chemotherapy Use and Outcomes in Solid Tumors: a systematic review. J Clin Oncol. 2011 Jan;29(1):106–17. 10.1200/JCO.2010.31.3049 21098314

[23] Hsieh M-C, Thompson T, Wu X-C, Styles T, O’Flarity MB, Morris CR, et al. The effect of comorbidity on the use of adjuvant chemotherapy and type of regimen for curatively resected stage III colon cancer patients. Cancer Med. 2016 May;5(5):871–80. 10.1002/cam4.632 PMC4864816 26773804

[24] Edwards MJ, Campbell ID, Lawrenson RA, Kuper-Hommel MJ. Influence of comorbidity on chemotherapy use for early breast cancer: systematic review and meta-analysis. Breast Cancer Res Treat. 2017 Aug;165(1):17–39. 10.1007/s10549-017-4295-4 28528451

[25] Stavrou EP, Lu CY, Buckley N, Pearson S. The role of comorbidities on the uptake of systemic treatment and 3-year survival in older cancer patients. Ann Oncol. 2012 Sep;23(9):2422–8. 10.1093/annonc/mdr618 22351742

[26] Nilsson J, Berglund A, Bergström S, Bergqvist M, Lambe M. The role of comorbidity in the management and prognosis in nonsmall cell lung cancer: a population-based study. Acta Oncol. 2017 Jul;3(7):949–56. 10.1080/0284186X.2017.1324213 28486004

[27] Janssen-Heijnen MLG. Effect of comorbidity on the treatment and prognosis of elderly patients with non-small cell lung cancer. Thorax. 2004 Jul 1;59(7):602–7. 10.1136/thx.2003.018044 PMC1747080 15223870

[28] Piccirillo JF. Prognostic importance of Comorbidity in a hospital-based Cancer Registry. JAMA. 2004 May;26(20):2441. 10.1001/jama.291.20.2441 15161894

